# Concilience in Entomopathogenic Nematode Responses to Water Potential and Their Geospatial Patterns in Florida

**DOI:** 10.3389/fmicb.2016.00356

**Published:** 2016-03-31

**Authors:** Fahiem El-Borai, Nabil Killiny, Larry W. Duncan

**Affiliations:** ^1^Citrus Research and Education Center, University of FloridaLake Alfred, FL, USA; ^2^Plant Protection Department, Faculty of Agriculture, Zagazig UniversityZagazig, Egypt

**Keywords:** conservation biological control, entomopathogenic nematodes, soil water potential, proteins

## Abstract

The geospatial patterns of four species of native entomopathogenic nematodes in Florida were previously shown to be related to soil properties that affect soil water potential. Here we compared the responses to water potential of third stage, infective juvenile (IJ), *Steinernema* sp. (Sx), and *Steinernema diaprepesi* (Sd) in controlled conditions. The two species were selected because they are closely related (*Steinernema glaseri*-group), but tend to occupy different habitats. In columns of sandy soil with moisture gradients ranging from field capacity (6% w:w) to saturated (18%), Sx migrated toward wetter soil whereas Sd migrated toward drier soil. Survival of two isolates each of Sx and Sd for 7 days in the absence of food was greatest at 18 and 6% soil moisture, respectively. After three cycles of migration through soil to infect insect larvae 10 cm distant, Sd dominated EPN communities when soil columns were maintained at 6% moisture, whereas Sx was dominant in soil maintained at 18% moisture. When rehydrated after 24 h on filter paper at 90% RH, 50% of Sd survived compared to no Sx. Two isolates of Sd also survived better than two isolates of Sx during up to 24 h in a hypertonic solution (30% glycerol). The behavioral responses of both species to water potential and osmotic gradients were consistent with surveys in which Sx was recovered only from flatwoods ecoregions with shallow water tables and poorly drained soils, whereas Sd most frequently inhabited the central ridge ecoregion comprising well-drained soils and deeper water tables. Comparative proteomic analysis revealed differential expression of proteins involved in thermo-sensation (guanylyl cyclase and F13E6-4) and mechano-sensation and movement (paramyosin, Actin 3, LET-99, CCT-2), depending on whether Sd was in soil at 6 or 18% moisture. Proteins involved in metabolism, lectin detoxification, gene regulation, and cell division also differed between the two conditions. Our data suggest the plausibility of modifying soil moisture conditions in flatwoods orchards in ways that favor more desirable (effective) EPN species. Similarly, these particular behavioral traits are likely to be useful in guiding the selection or engineering of EPN species for use in different ecoregions.

## Introduction

Florida citrus orchards are inhabited by diverse and abundant communities of native entomopathogenic nematodes (EPNs) that contribute significantly to the biological control of soilborne insect pests (Beavers et al., 1983; McCoy et al., 2000; Duncan et al., 2003, 2007). These nematodes, in the genera *Steinernema* and *Heterorhabditis*, infect insect larvae and then release symbiotic bacterial entomopathogens that kill the insect. Of nine EPN species reported to inhabit citrus orchards and adjacent natural areas in Florida, four are encountered most frequently. *Heterorhabditis indica* Poinar, Kanunakar, and David is virtually ubiquitous in Florida orchards, while other species are more restricted in the habitats they occupy (Duncan et al., 2003, 2007, 2013; Campos-Herrera et al., 2013a,c). Orchards on the central ridge usually support populations of *Steinernema diaprepesi* Nguyen and Duncan and often *Heterorhabditis zealandica* Poinar. An undescribed *Steinernema* sp. *glaseri*-group is found occasionally in orchards in the various flatwoods regions where *S. diaprepesi* and *H. zealandica* are infrequently encountered. The central ridge is an ecoregion characterized by higher elevation and deep, well-drained sandy soil in contrast to flatwoods regions which are at lower elevation and have shallow water tables and finer textured sandy soils that tend to be less well-drained than those of the central ridge.

The abundance of the root weevil pest *Diaprepes abbreviatus* L. is generally greatest in poorly drained areas of flatwoods orchards and least in well-drained soils on the central ridge (McCoy, 1999). The potential role of EPNs in modulating weevil population density is poorly understood. EPN communities tend to be less species rich and diverse in habitats that favor large *D. abbreviatus* populations, compared to habitats with fewer weevils, but EPN population size does not appear to differ greatly between those habitats (Campos-Herrera et al., 2013a). Mechanisms other than EPN population density that might enhance EPN regulation of weevils on the central ridge compared to the flatwoods include better efficacy in the more porous, central ridge soils (Campos-Herrera and Gutiérrez, 2009), greater EPN species diversity and richness (Jabbour et al., 2011), and/or the occurrence of more effective EPN species (Gaugler, 1999). Greenhouse experiments indicated that *S. diaprepesi* and *Steinernema* sp. reduced weevil feeding damage to citrus roots more than did either *H. indica* or *H. zealandica* (El-Borai and Duncan, 2007; El-Borai et al., 2012). Widespread occurrence of *S. diaprepesi* in central ridge orchards is consistent with lower weevil numbers there. However, the infrequent occurrence of *Steinernema* sp. in flatwoods orchards may favor larger weevil populations in EPN-depauperate areas.

The abundant, but regionally distinctive EPN communities in Florida orchards suggest the possibility of developing conservation biological control tactics to modify less conducive habitats in ways that make them more favorable to certain EPN species. Moreover, the difference in habitats occupied by two closely related species in the *S. glaseri*-group V (Spiridonov et al., 2004; Campos-Herrera et al., 2011), suggests the potential utility of *S. diaprepesi* and *Steinernema* sp. as models to study habitat adaptation. Campos-Herrera et al. (2013a) analyzed the spatial patterns of EPNs and more than 30 biotic and abiotic properties of soils in citrus orchards across the Florida peninsula. Four soil properties that affect soil water potential (water holding capacity, organic matter, clay content and depth to ground water) explained the most variability in EPN communities. Accordingly, in the present study we compared the responses of *S. diaprepesi* and *Steinernema* sp. to a variety of conditions with water potentials similar to those encountered in the central ridge (from dry to field capacity) and flatwoods (from dry to saturated) soil habitats in Florida. Our hypothesis was that *S. diaprepesi* and *Steinernema* sp. would respond positively to drier and wetter conditions, respectively. We also characterized differences in protein expression by *S. diaprepesi* in wetter and drier soil conditions in order to identify proteins that are potentially involved in the adaptation to soil water potential.

## Materials and methods

### Entomopathogenic nematode cultures

*Steinernema diaprepesi* (Sd), *Steinernema* sp. *glaseri*-group (Sx), *Heterorhabditis zealandica* (Hz), and *H. indica* (Hi) were originally isolated from caged *D. abbreviatus* larvae buried for several days in commercial citrus orchards in Florida. Morphological and molecular analyses were performed for identification upon collection (Nguyen, 2007; Campos-Herrera et al., 2011). Several geographic isolates of all EPN species were maintained in pure cultures in the laboratory using the last instar larvae of the greater wax moth, *Galleria mellonella* L. (Woodring and Kaya, 1988). Infective juveniles (IJs) that emerged from insect cadavers into White traps were harvested in tap water and stored at 15°C for 1–5 days before use. The isolates Sd Hancock (GPS coordinates and Genbank accession number; 28.293232, −82.257191, GU173996) and Sx Webber (28.2526, −82.4741; not submitted) were used in all experiments. Two additional isolates, Sd Bartow (27.885035, −81.756878, GU173994) and Sx Arcadia (27.2273, −81.9649, GU174002), were used to further test and validate species differences in some experiments described below. The *S. diaprepesi* isolates were collected from sites 80 km distant from each other and those of Sx were separated by 122 km.

### EPN migration in moisture gradients

Sand columns were constructed as described by El-Borai et al. (2011) with the modification of using a 3.5 cm length of Tygon® tubing to tightly connect two glass jars (17 cm^3^ volume each; BTL, sample type 111, CLR, SNAPC; Wheaton Science Products, Millville, NJ.). One jar was filled with sand at 6% moisture (wt water/dry wt soil) and the other jar was filled with sand at 18% moisture. The connecting tube was filled with sand at 10% moisture. All parts were connected together and 200 IJs of Sd or Sx in 0.2 ml water were introduced into the center of the column through a small hole in the Tygon tubing. The sand used in all experiments was obtained by washing and autoclaving a sandy soil through a 40 mesh sieve (0.425 mm openings) onto a 100 mesh sieve (0.150 mm openings) where it was washed repeatedly. The resulting clean sand facilitated recovery of test nematodes by rinsing the substrate from jars into 100 mL water, swirling the suspension, permitting the soil to settle for 3–4 s, and decanting the nematodes for counting (Abou-Setta and Duncan, 1998). Soil columns were arranged in a plastic box lined with moist paper with equal numbers of jars containing 6 or 18% soil moisture pointing in the same cardinal direction. After 24 h the columns were disassembled and nematodes from each jar were recovered and counted. Two experiments were conducted, each with 15 replications. Paired *t*-tests were used to determine whether the number of recovered IJs differed significantly from one soil moisture to the other.

### EPN survival and soil moisture in the absence of host insects

The experimental units consisted of sterile Petri dishes (60 × 15 mm) filled with soil obtained from two treatments in an ongoing field experiment near Auburndale on the Florida central ridge (81:48:44.65W, 28:06:53.98N, 49 m elevation). Soil from an “advanced production system” (APS) treatment designed to grow citrus trees more quickly was fertigated daily for 2 years via drip irrigation (Schumann et al., 2012). Soil from a conventional citriculture treatment was fertilized with dry fertilizer four times annually and irrigated as needed by microsprinklers. The APS treatment changed several soil properties compared to those under conventional citriculture and reduced the abundance of naturally occurring Sd in the field plots (Campos-Herrera et al., 2013c, 2014). The soil was either oven dried (70°C) in one trial and autoclaved and air dried before used in a second trial. The moisture content (g water per 100 g dry soil) of the soil was adjusted to 2% (very dry), 6% (field capacity), and 18% (saturated) by mixing with tap water. Approximately 1300–1400 IJs in 0.5 ml of tap water were pipetted onto the soil surface of each plate. The moisture level adjustments were calculated to include the water in the nematode inoculum. Twenty replications for each EPN species/soil moisture/soil type were prepared. All plates were sealed with parafilm and incubated at 27°C. After 7 days, soil and inner dish surfaces of each plate were rinsed into Baermann funnels and IJs were recovered after 1 week. In a second trial, the nematode extraction was done directly using sucrose centrifugation (Jenkins, 1964). All live IJs were counted with the aid of an inverted compound microscope (20–400 ×). Treatment effects within each species were determined by two-way ANOVA (cultural practice x moisture level) followed by Tukey's HSD-test (*P* = 0.05). The experiment was conducted twice.

Sandy soil from a citrus orchard at the Citrus Research and Education Center in Lake Alfred was autoclaved, air-dried, and used to test the survival of two isolates each of Sd and Sx at soil moistures of 6 and 18%. The experiment was conducted as described previously with Baermann funnels used to recover IJs.

### EPN community structure and soil moisture in presence of host insects

Petrie dish assays at soil moistures of 6 and 18% were conducted as described in the previous section except that air dried Candler sand (97:2:1, sand:silt:clay) soil was used and a mixture of 500 IJs each of Sd, Sx, Hi, and Hz were added to each dish. Treatments were replicated 30 times. After 7 days, IJs were extracted from half of the assay dishes per treatment as described above. The other half were inverted and placed on the top of soil columns contained in a PVC tube (10 × 5-cm-diameter). The soil moisture in each column was the same (6 or 18%) as that of the soil in the inverted Petrie dish. The columns were wrapped in aluminum foil and maintained at room temperature (23 ± 3°C) for 14 days, after which they were secured with duct tape to the top of a second, identical soil column containing four *G. mellonella* larvae. Fly screen (2 mm openings) was fastened with glue to the bottom of each column section to secure the larvae within the bottom section. The sand columns were again covered with aluminum foil and maintained for two additional weeks after which they were disassembled. The bottom section was secured to the top of an identical column containing four *G. mellonella* larvae and the IJs in the top section were recovered using sucrose centrifugation (Jenkins, 1964) and counted with an inverted compound microscope to facilitate species determination. The community composition in the top section at this point was considered the first “generation.” The process was repeated twice more (where survivors in the bottom section were used to initiate a new round of competition) to produce three experimental generations during which the species competed with one another in two soil moisture conditions. For each generation and moisture level, the proportion of the total EPN population of each species was compared by ANOVA and differences were separated by Tukey's HSD.

### Relative humidity, hypertonicity, and EPN survival

Glass specimen jars (150 ml volume, 5 cm diameter) containing 115 ml solution of 15, 30, and 80% (wt:wt) glycerin in water produced an estimated ambient relative humidity of 95, 90% and 50%, respectively (Foruney and Brandl, 1992). Approximately 2000 IJs of Sd or Sx in 50 μl water were pipetted onto each of three filter paper strips (2 × 0.5 cm) contained in the lids of Petrie dishes (3.8 cm diameter) that were floated on the solutions in the sealed jars at room temperature. After 24 h strips were placed into water in individual Petrie dishes where IJs rehydrated for 24 h before being counted as dead (non-motile) or alive. Non-motile specimens were touched with an eyelash probe to determine whether they responded with movement. The experiment was repeated once.

Aliquots of ca. 2000 IJs of two isolates each of Sd and Sx were also placed directly into solutions of 30% glycerol in 8-ml sample bottles (Wheaton, Corp. Millville, NJ). After 12, 18, and 24 h, three bottles containing each nematode isolate were poured onto 38-mm opening sieves and backwashed into counting dishes. After 24 h rehydration, IJ motility was evaluated as described above. In the first trial, the isolates Sd Hancock and Sx Webber were compared with one another. In a second trial Sd Bartow and Sx Arcadia were used. Data were expressed as percent survival and were transformed to arcsin-square root before analysis of variance at each time point followed by Tukey's HSD-test for mean separation. In a third trial, all four isolates were compared together in the same experiment.

### Protein expression at different soil moistures

Washed sand was prepared as previously described and soil moisture adjusted to 6% or 18%. Approximately 1300–1400 IJs of either Sd or Sx in 0.5 ml of distilled water were pipetted onto the soil surface of each plate and 20 replicate plates of each treatment were sealed and maintained as previously described. After 48 h IJs were recovered by rinsing and decanting as previously described. IJs were further separated from soil debris by centrifugation in a MgSO_4_ gradient (Kaplan and Davis, 1990). IJs were then incubated with four volumes of ice-cold acetone at −20°C overnight, after which the proteins were further cleaned with 2-D Clean-up Kit (GE) following the manufacturer's instructions. Sd and SX proteins were dissolved in rehydration buffer [7 M urea, 2 M thiourea, 60 mM dithiothreitol (DTT), 65 mM 3-(3-cholamidopropyl dimethylammonio)-1-propanesulfonic acid (CHAPS), 2% Trion X-100, 0.2% ampholytes 5–8] and briefly sonicated. After a 14,000 g centrifugation at ambient temperature for 30 min, the protein concentration of the supernatant was determined by the Bradford method (Bradford, 1976). For the first dimension of isoelectric focusing (IEF), 350 μg of solubilized proteins was dissolved in 300 μl of rehydration buffer with trace bromophenol blue dye, and loaded onto a 17-cm immobilized pH gradient (IPG) linear pH 5–8 strip (Bio-Rad). After an active rehydration step at 20°C for 13 h at 50 V, the strips were automatically focused using the following parameters: 250 V, linear, 1 h; 500 V, slow, 1 h; 1000 V, slow, 1 h; 5000 V, slow, 1 h; 10,000 V, linear, 3 h; 10,000 V, rapid, 70,000 Vh (Lu et al., 2010). The current limit for each strip was set to 50 μA. After IEF, the strips were first incubated in equilibration buffer (6 M urea, 20% glycerol, 2% SDS and 0.375 mM Tris–HCl, pH 8.8) containing 2% (w/v) DTT for 15 min with gentle shaking, then a second equilibration in equilibration buffer containing 2.5% (w/v) iodoacetamide instead of DTT for 15 min. Equilibrated IPG strips were further resolved with 9.5% SDS-PAGE gels (1.5 mm gel thickness), and the program was 5 mA/gel for 40 min, then 30 mA/gel until the bromophenol blue dye front reached the bottom of the gel. Each treatment was resolved with 2-DE at least three times to obtain reliable and statistically significant results. The gels were stained following a modified Colloidal Coomassie G-250 staining protocol (Dyballa and Metzger, 2009). The stained gels were scanned and imported into Melanie 7 software for various analyses including spot detection, matching, and quantitative intensity analysis. Three independent experiments were performed to validate the results. Only those unique or significantly different protein spots (*P* < 0.05) were selected and subjected to identification by mass spectrometry (MS).

LC-MS/MS was done in the Interdisciplinary Center for Biotechnology Research (ICBR), University of Florida, Gainesville. The protein was reduced, alkalated in-gel, and digested with trypsin (Promega) at 37°C overnight. The enzymatically digested samples were injected onto a capillary trap (LC Packings PepMap) and desalted for 5 min with a flow rate 3 μl/min of 0.1% (v/v) acetic acid. The samples were loaded onto an LC Packing® C18 Pep Map nanoflow HPLC column. The elution gradient of the HPLC column started at 3% solvent A (0.1% v/v acetic acid, 3% v/v ACN, and 96.9% v/v H_2_O), 97% solvent B (0.1% v/v acetic acid, 96.9% v/v ACN, and 3% v/v H_2_O), and finished at 60% solvent A, 40% solvent B for 60 min for protein identification. LC-MS/MS analysis was carried out on a LTQ Orbitrap XL mass spectrometer (ThermoFisher Scientific, West Palm Beach, FL). The ion spray voltage was set to 2200 V. Full MS scans were acquired with a resolution of 60,000 in the orbitrap from m/z 300 to 2000. The five most intense ions were fragmented by collision induced dissociation (CID). Dynamic exclusion was set to 60 s. Tandem mass spectra were extracted. All MS/MS samples were analyzed using Mascot (Matrix Science, London, UK; version 2.4.0). Mascot was set up to search NCBI_other Metazoa databases assuming the digestion enzyme trypsin. Mascot was searched with a fragment ion mass tolerance of 0.8 Da and a parent ion tolerance of 10 ppm. Iodoacetamide derivative of Cys, deamidation of Asn and Gln, oxidation of Met, are specified in Mascot as variable modifications. Scaffold (version Scaffold-4.0, Proteome Software Inc., Portland, OR) was used to validate MS/MS based peptide and protein identifications. Peptide identifications were accepted if they could be established at >95.0% probability as specified by the Peptide Prophet algorithm (Keller et al., 2002; Nesvizhskii et al., 2003). Protein identifications was accepted if they could be established at >95.0% probability and contain at least two identified unique peptides.

## Results

### EPN migration in moisture gradients

**Figure 1:**
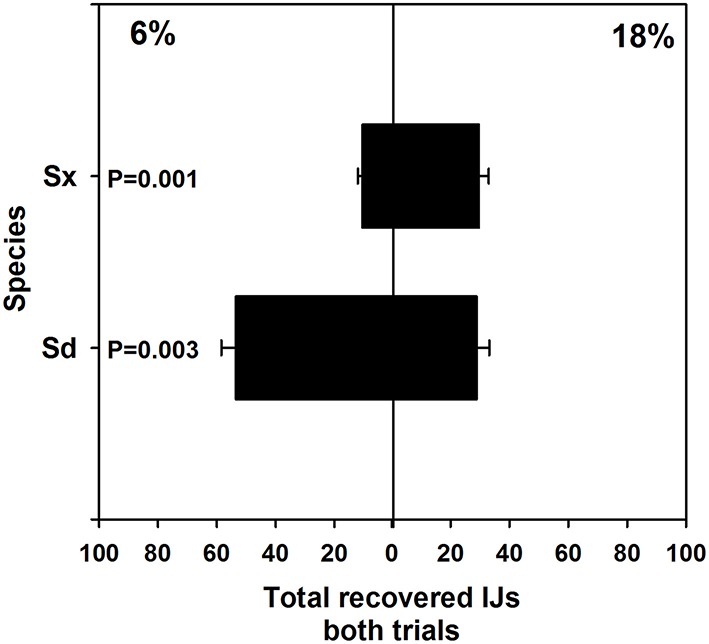
**Mean (and standard error) number of infective juvenile ***Steinernema*** sp. and ***S. diaprepesi*** recovered from the field capacity (6%) or saturated (18%) ends of soil columns with moisture gradients**. Nematodes were recovered 24 h after placement in the center of the horizontal sand columns. Sd, *Steinernema diaprepesi*, Sx, *Steinernema* sp.

Pooled results from two experiments revealed that Sd and Sx migrated preferentially toward drier and wetter conditions, respectively (Figure 1). Sixty-five percent of the recovered Sd (*P* = 0.003) and 61% of the recovered Sx (*p* = 0.001) migrated toward their preferred moisture levels.

### EPN survival and soil moisture in the absence of host insects

**Figure 2:**
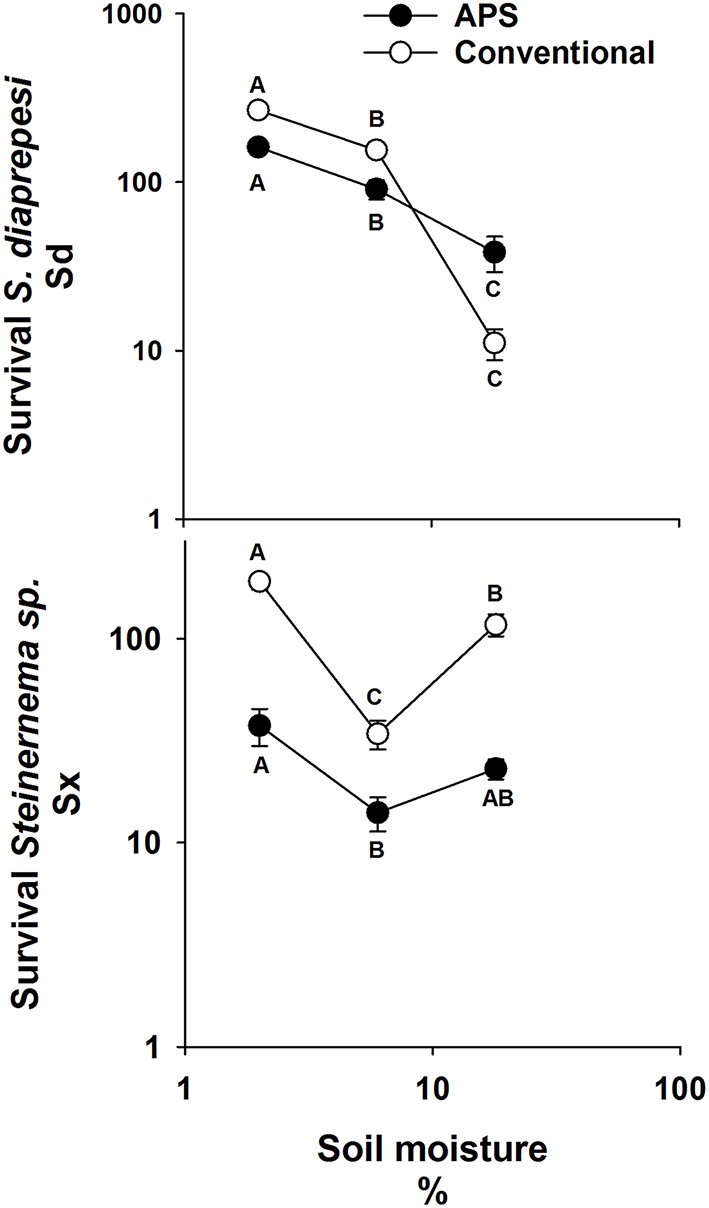
**Mean (and standard error) number of infective juvenile ***Steinernema*** sp. and ***S. diaprepesi*** recovered following 7 d of storage in field capacity (6%) or saturated (18%) sand that originated from experimental field plots managed with conventional or advanced citriculture methods**. Data shown on log scaled axes. Points on the same curve with the same letters do not differ significantly (*P* > 0.05) according to Tukey's HSD-test. Sd = *Steinernema diaprepesi*, Sx = *Steinernema* sp.

Significantly more of both EPN species survived in soil managed by either cultural practice (APS or CC) when soil moisture was 2% than when it was wetter (Figure 2). Regardless of cultural practice, five-fold more Sd survived at 6% than at 18% moisture (*P* = 0.01), whereas just 34% as many Sx survived at 6% compared to survival at 18% (*P* = 0.01). Survival of Sd was significantly lower in APS soil than in that of CC when moisture was at 2 and 6%. Survival of Sx was also lower in APS compared to CC soil when moisture was at 2% and at 18%. The different soils did not affect survival of either species at the soil moisture least favorable for its survival.

**Figure 3:**
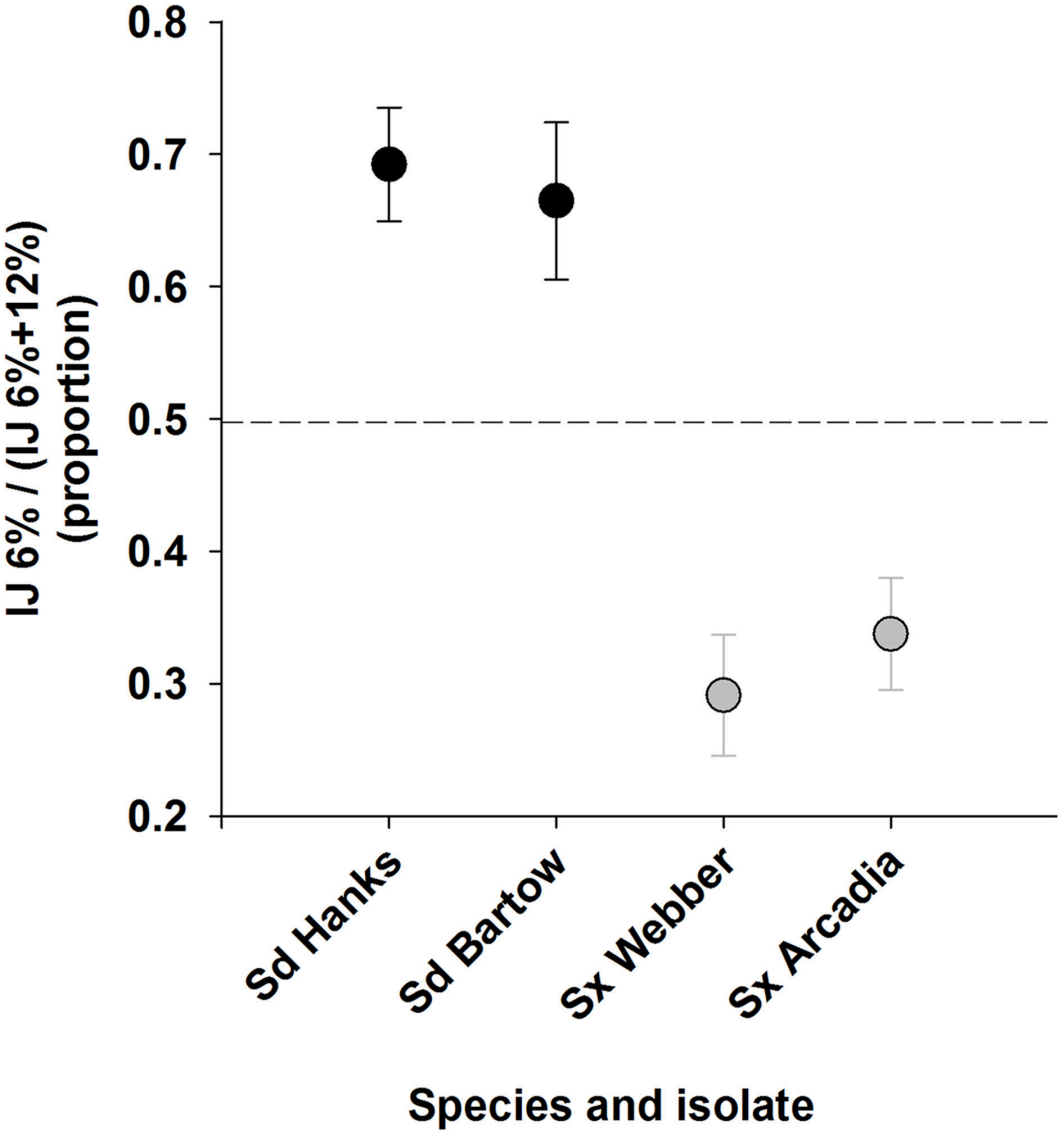
**Recovery of two isolates each of infective juvenile ***Steinernema*** sp. and ***S. diaprepesi*** recovered following 7 d of storage in field capacity (6%) or saturated (18%) sand that originated from a citrus orchard**. Proportions were calculated as numbers of nematodes recovered from sand at field capacity divided by the total number of nematodes recovered from soil at both soil moistures. Sd = *Steinernema diaprepesi*, Sx = *Steinernema* sp.

Two isolates each of Sd and Sx responded consistently to maintenance at 6% or 18% soil moisture (Figure 3). Approximately two-thirds of the total Sd IJs were recovered from the dryer soil and a similar proportion of Sx IJs were recovered from the wetter soil. The proportion recovery of each species from the two soil conditions differed from 50% (*P* < 0.001) and there were no significant differences in response to moisture between the two isolates of either species.

### EPN community structure and soil moisture in presence of host insects

**Figure 4:**
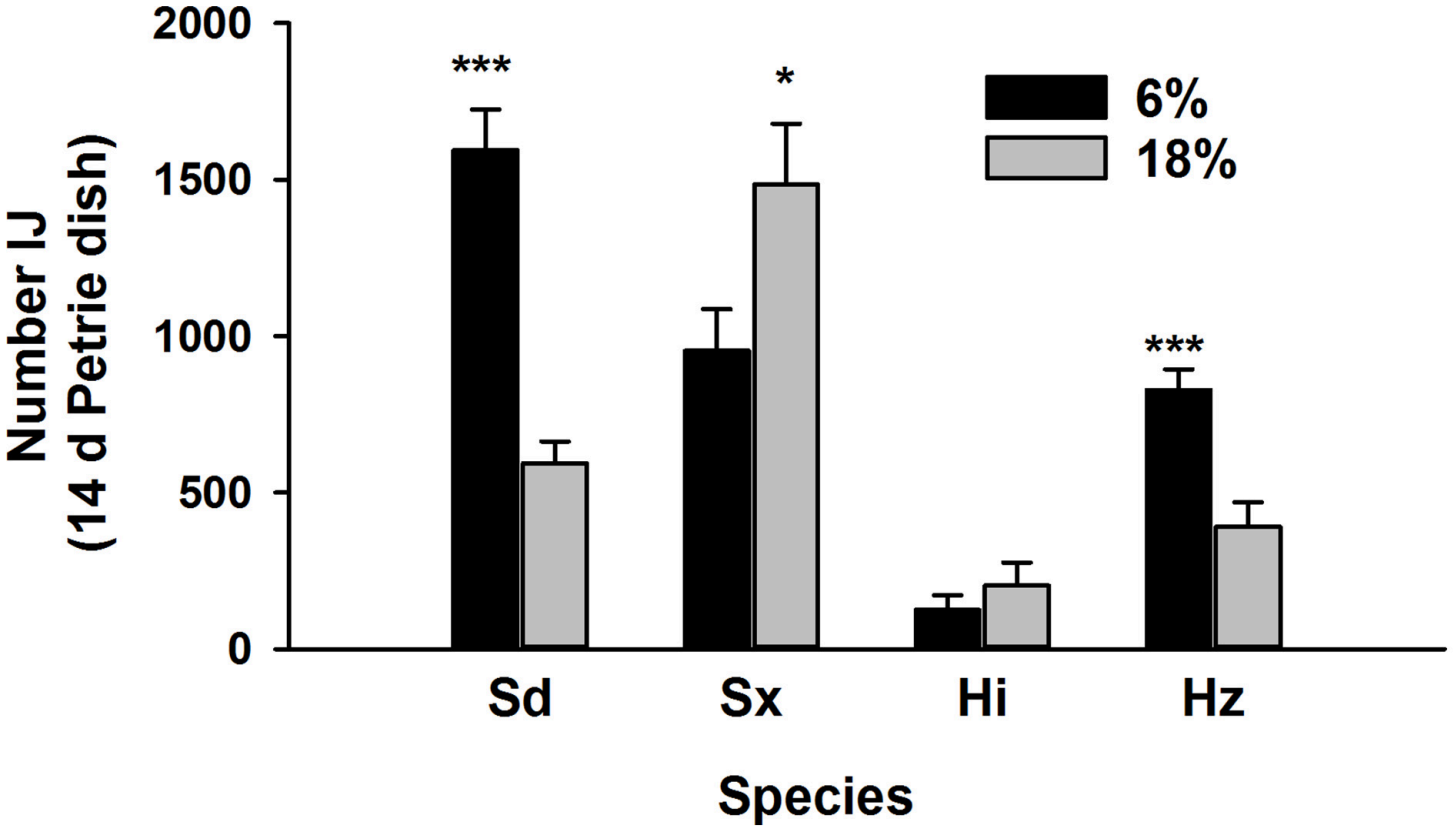
**Recovery of four species of entomopathogenic nematodes (Sd = ***Steinernema diaprepesi***, Sx = ***Steinernema*** sp., Hi = ***Heterorhabditis indica***, Hz = ***H. zealandica***) after 14 days in Petrie dishes filled with sand moistened to field capacity (6%) or saturated (18%)**. Significant differences in recovery from each moisture for each species denoted by ^***^P < 0.001 and ^*^P < 0.05.

**Figure 5:**
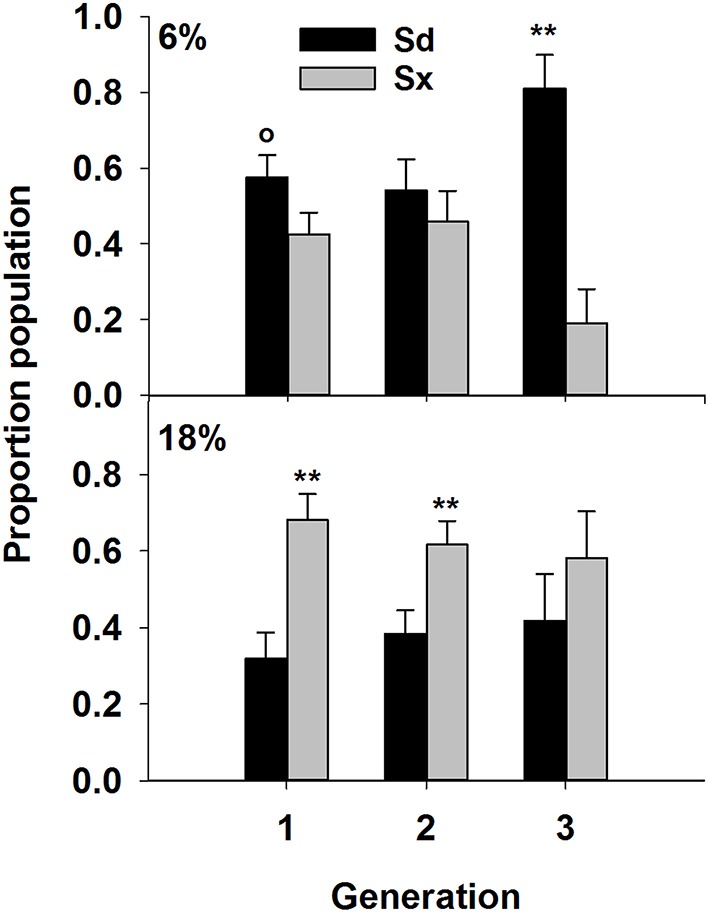
**Proportion of the total nematode population represented by Sd (***Steinernema diaprepesi***), or Sx (***Steinernema*** sp.) for three “generations” of nematodes maintained with ***Galleria mellonella*** larve in PVC columns filled with sand at field capacity (6% moisture) or saturated (18%)**. Significant differences between species within a generation from each moisture for each species denoted by ^**^P < 0.01 and °*P* < 0.10.

Nematode survival after 7 days in the Petrie dishes was similar to previous results (Figure 4). Survival of Sd and Sx was greatest at 6% (*P* = 0.001) and 18% (*P* = 0.05), respectively. Fewer Hi survived than other species and soil moisture had no effect on the species. The 6% treatment favored survival of Hz (*P* = 0.001). The three subsequent generations contained communities of just Sd and Sx (Figure 5). In two of the three generations, Sd was the dominant community member in soil of 6% moisture and Sx dominated communities in soil at 18% moisture.

### Relative humidity, hypertonicity, and EPN survival

**Figure 6:**
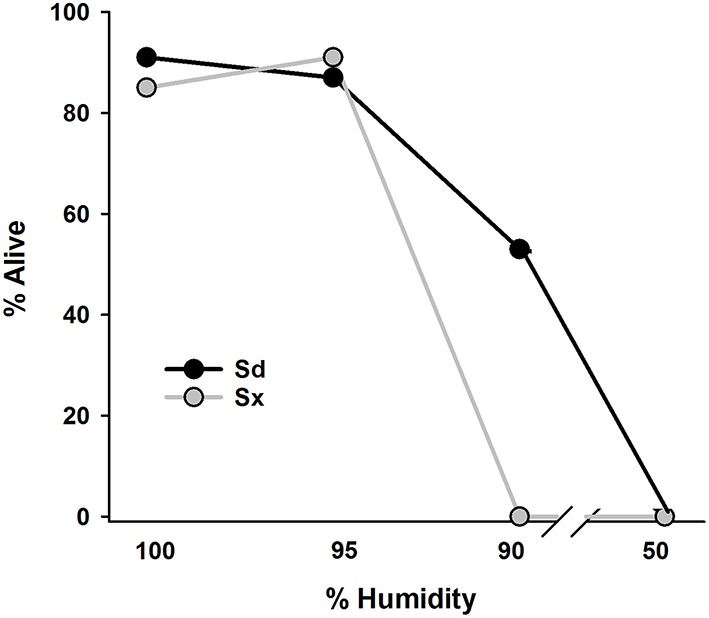
**Percentage of surviving infective juvenile Sd (***Steinernema diaprepesi***) or Sx (***Steinernema*** sp.) after 24 h storage at 100, 95, 90, or 50% relative humidity**. Humidity conditions were created by floating Petrie dish lids containing filter paper and EPNs on either tap water or glycerol solutions in sealed containers.

Survival of both EPN species after 24 h on filter paper was >80% when incubated at 95% and 100% RH and none of either species survived at 50% RH (Figure 6). At 90% RH, about half of the Sd and none of the Sx remained motile.

**Figure 7:**
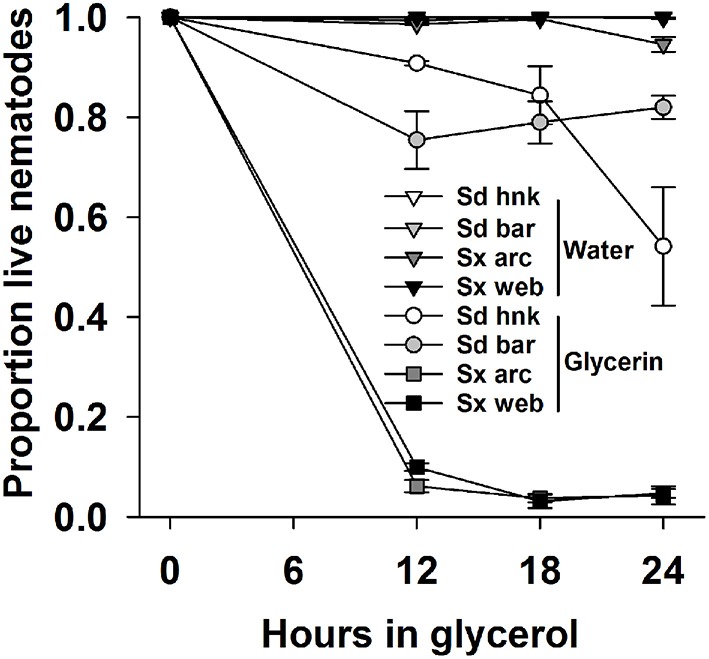
**The proportion survival of two isolates each of Sd (***Steinernema diaprepesi***) and Sx (***Steinernema*** sp.) stored for 12, 18, or 24 h in water or 30% glycerin solution, then rinsed and stored for 24 h in water**. Error bars are SEM.

When incubated in 30% glycerin solution, both Sd isolates survived at higher rates than either isolate of *Steinernema* sp. (Figure 7). All IJs were flattened, distorted and apparently completely desiccated upon removal from the glycerin. All appeared normally rehydrated after 24 h recovery in water; however dead IJs were internally disorganized. Survival for both isolates of Sx ranged between 2 and 10% after 12 and 24 h in glycerin with virtually no mortality of IJs in water. The survival of Sd IJs isolated from Bartow remained above 75% throughout the experiment. The Sd IJs isolated from Hancock behaved similarly until mortality increased to about half after 24 h. Both isolates of Sd survived better (*P* = 0.001) than either isolate of Sx. Results of the experiments when repeated were essentially the same.

### Sd protein expression at different soil moistures

**Figure 8:**
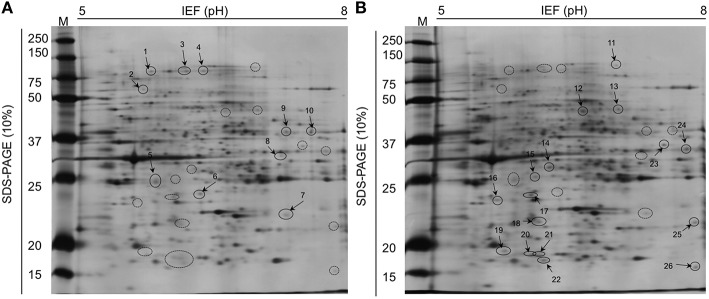
**Two-dimensional gel electrophoresis comparisons of total proteins isolated from ***Steinernema diaprepesi*** maintained for 48 h in sand at 6% moisture (A) and 18% moisture (B)**. IPG strips of linear pH 5–8 were used for isoelectric focusing and the SDS-PAGE was performed in 10% acrylaMIDE. Protein molecular weight standards are shown on the left. Spots of statistically significant differences (*P* < 0.05) between the condition were selected to be identified using mass spectrometry and listed in Table 1. Spots labeled 1–10 were proteins expressed at highest levels in 6% moisture sand, those from 11 to 26 were expressed at higher levels in 18% moisture sand.

Twenty-six proteins occurred in different amounts in IJs, depending on soil moisture conditions (Figure 8). Ten proteins were detected at higher concentration and 16 proteins at lower concentration in IJs stored in soil at 6% moisture for 24 h compared to IJs stored at 18% moisture. Histone 67 and Actin 3 were present in different forms at higher and lower levels in IJs from both soil moisture conditions. Differential expression occurred for proteins involved in thermo-sensation (guanylyl cyclase and F13E6-4) and mechano-sensation and movement (paramyosin, Actin 3, LET-99, CCT-2). Proteins involved in metabolism, lectin detoxification, gene regulation and cell division were also among those that differed between the two conditions.

## Discussion

The responses of Sd and Sx to different hydraulic and osmotic conditions were consistent with their natural geospatial patterns and with the hypothesis that they are physiologically adapted to drier and wetter soil conditions, respectively. Presumably, Sd is adapted to drier conditions in the central ridge than is Sx, in part due to a superior ability for osmoregulation and desiccation survival. Conversely, the attraction to moist soil exhibited by Sx and its ability to persist better than Sd in wetter conditions suggest the occurrence of adaptive behaviors that were not addressed in this study. These experimental results lend support to the causative nature of correlations reported between variables that modulate soil moisture and the occurrence of EPNs in Florida orchards (Campos-Herrera et al., 2013a).

**Table 1:** **Differentially-expressed proteins from ***Steinernema diaprepesi*** grown in sand at 6% or 18% moisture, as identified by LC-MS/MS**.

	**No.^a^**	**Protein name^b^**	**NCBI Accession**	**Fold change^c^ [(18–6%)/6%]**	**Coverage^d^(%)**	**Matched unique peptides^d^**	**Protein identification probability^d^(%)**	**Molecular and biological function**	**Known role in nematode**
Structural proteins (muscles/movement)	1	Paramyosin	gi|127760	–4	20	20	100	Major component of thick filaments in many invertebrate	Necessary for determination of nematode thick filament length *in vivo* (Mackenzie and Epstein, 1980)
Mechanical response	2	Paramyosin	gi|127760	S6%	20	24	100	Major component of thick filaments in many invertebrate	Necessary for determination of nematode thick filament length *in vivo* (Mackenzie and Epstein, 1980)
	13	ACT-3 (Actin 3)	gi|14278	2.2	5	5	100	The actin cytoskeleton drives the cellular rearrangements underlying morphogenesis, through regulated polymerization or actomyosin contraction.	
	28	LET-99	gi|17541472	–1.8	1.3	1	99	LET-99 is a DEP (Disheveled/EGL/Pleckstrin) domain protein (nematode specific)	Required for the proper orientation of spindles after the establishment of polarity (Tsou et al., 2002).
									^*^no homolog in non-nematode
	26	CCT-2 (Chaperonin containing T-complex polypeptide 1 subunit beta)	gi|1046266	7.5	2	1	100	Known to play a role, *in vitro*, in the folding of actin and tubulin	
	10	ACT-3 (Actin 3)	gi|14278147 NP_505817	–5.2	15	2	100	The actin cytoskeleton drives the cellular rearrangements underlying morphogenesis, through regulated polymerization or actomyosin contraction.	
Gene regulation/cell division	3	HIS-44 (Histone 44)	gi|17532989	–1.7	20	3	100	One of the five main histone proteins involved in the structure of chromatin in eukaryotic cells. Featuring a main globular domain and a long N terminal tail H2B is involved with the structure of the nucleosomes of the ‘beads on a string’ structure	
	16	HIS-67(Histone 67)	gi|17509199	3.1	17	2	100	One of the five main histone proteins involved in the structure of chromatin in eukaryotic cells. Featuring a main globular domain and a long N terminal tail H2B is involved with the structure of the nucleosomes of the ‘beads on a string’ structure	
	14	HIS-67(Histone 67)	gi|17509199	S18%	26	3	100	One of the five main histone proteins involved in the structure of chromatin in eukaryotic cells. Featuring a main globular domain and a long N terminal tail H2B is involved with the structure of the nucleosomes of the ‘beads on a string’ structure	
	4	HIS-67(Histone 67)	gi|17509199	–4.2	29	3	100	One of the five main histone proteins involved in the structure of chromatin in eukaryotic cells. Featuring a main globular domain and a long N terminal tail H2B is involved with the structure of the nucleosomes of the ‘beads on a string’ structure	
	7	EEF-2, isoform a	gi|156279	–1.3	5	7	100	Main regulator of peptide chain elongation in eukaryotic cells.	
	8	EEF-2, isoform a	gi|156279	6%		8	100	Main regulator of peptide chain elongation in eukaryotic cells.	
	9	AKT-1, isoform a	gi|71983636	–7	2	1	98	Regulator of apoptosis in limiting cytokine concentrations	
	11	CYN-4 (Peptidyl-prolyl cis-trans isomerase 4)	gi|17532641	1.3	2.5	1	97	Required for body wall muscle cell development.	
Thermal response	6	GCY-6 (Guanylyl Cyclase)	gi|453232464	S6%	1	1	98	Guanylyl cyclase synthesizes cGMP from GTP in response to calcium level	Thermosensory receptor only in left neuron in terms of lateral bisymetry in *C. elegans* (Inada et al., 2006)
	27	F13E6-4	gi|71987524		2	1	97	F13E6.4 gene encodes a protein that shows sequence similarities to YAP	Involved in the thermotolerance and aging in *C. elegans*. (Iwasa et al., 2013)
	20	F13E6-4	gi|71987524	3.5	2	1	95	F13E6.4 gene encodes a protein that shows sequence similarities to YAP	Involved in the thermotolerance and aging in *C. elegans*. (Iwasa et al., 2013)
	12	F13E6-4	gi|71987524	S18%	2	1	91	F13E6.4 gene encodes a protein that shows sequence similarities to YAP	Involved in the thermotolerance and aging in *C. elegans*. (Iwasa et al., 2013)
metabolism	15	R12C12.1	gi|17535605	S18%	1.3	1	93	Glycine dehydrogenase	
	17	R12C12.1	gi|193208127	3.7	3.2	1	97	Glycine dehydrogenase	
	21	F46H5.3	gi|32566409	3.5	4.9	1	98	Arginine kinase	
	23	FUM-1	gi|17553882	1.4	6	2	100	Fumarase; converts fumaric acid to L-malic acid in the TCA cycle	
	22	MDH-2	gi|17554310	3.7	5	1	99	Methanol/ethanol family dehydrogenase	
Lectin detoxification	18	LEC-3	gi|17535117	2.9	4	2	100	32 kDa beta-galactoside-binding lectin lec-3	
	19	LEC-3	gi|17535117	S18%	3.8	1	99	32 kDa beta-galactoside-binding lectin lec-3	
	5	C08F11.7	gi|17538574	–2.1	4.1	1	98	Putative protein	

a *The spot numbers correspond to the numbers in 2DE gels in figure X*.

b *Proteins identification was done using C. elegans database*.

c *Quantitative analysis was performed with Melani 7 sofware*.

d *The three parameters of protein identification were generated using MASCOT software*.

Both survival and orientation by these two species were affected by soil moisture. The ability of each species to distinguish and migrate toward its favored water potential may have been detrimental for those IJs in their respective, least favorable treatment of the survival assay because those treatments were uniformly too wet (Sd at 18%) or too dry (Sx at 6%). Quiescence increases the survival rate of many nematode species by conserving energy reserves during commonly encountered, unfavorable soil conditions such as desiccation, anoxia, or host unavailability (reviewed by Evans and Perry, 2009). As for Sd and Sx in the present study, sandy soil at 2% and even lower moisture was shown previously to increase the survival rate of *Steinernema riobrave* compared to survival rates at higher moistures (Duncan et al., 1996; Duncan and McCoy, 2001). At such low water potential, the surface film of water on soil particles is unlikely to be thick enough to permit nematode motility, thereby inducing quiescence or even partial anhydrobiosis. However, where moisture is adequate for movement, as in the field capacity (6%) or saturated (18%) treatments, IJs may have responded to unsuitable water potentials with hyperactivity in search of more favorable conditions, thereby expending more energy and dying more quickly than IJs in more favorable conditions. Alternatively, if the dissolved oxygen concentration was reduced in the saturated compared to field capacity soils, differences in the capacity for anoxic quiescence may have caused the differences in survival rates of Sd and Sx (Kung et al., 1990).

The phylogenetic similarity of Sd and Sx, combined with their different phenotypic responses to water and osmotic potential, make them potentially useful to study environmental adaptation. The fundamental mechanisms of specific adaptive behaviors such as osmoregulation (Choe, 2013), anhydrobiosis (Erkut et al., 2013), and humidity sensation/orientation (Russell et al., 2014) are being increasingly resolved with respect to nematodes, primarily *Caenorhabditis elegans*. These findings are making it possible to study how suites of these behaviors are modulated by species for adaptation to specific habitats. For example, the different levels of guanylyl cyclase expressed by Sd in soil at 6 or 18% moisture may be indicative of responses to moisture variation. Russell et al. (2014) demonstrated that humidity is detected by *C. elegans* by interpreting thermos-sensory and mechano-sensory signals. Mutant worms deficient in guanylyl cyclases, required for thermosensation, were unable to perform hygrotaxis. Similarly, variation in levels of some of the proteins in this study involved in movement, metabolism and development may play roles in adaptive responses by Sd to variation in water potential. Further comparison of protein expression between populations of Sd and Sx that vary in their responses to moisture and hypertonic stress will help eventually to make the linkages between specific behaviors and habitat adaptation.

Regional geospatial patterns of EPN species are increasingly characterized, usually in conjunction with descriptive information about the sampled habitats (e.g., Mekete et al., 2005; Mwaitulo et al., 2011; Zadji et al., 2013; Valadas et al., 2014; Wang et al., 2014). Reports for a few commonly encountered species are consistent enough to speculate broadly about their biome preferences (see Hominick, 2002). More recently, surveys have been designed and analyzed to reveal associations between EPNs and specific edaphic properties that might affect EPN occurrence (Hoy et al., 2008; Kaspi et al., 2010; Kanga et al., 2011; Campos-Herrera et al., 2013a,b, 2016). This is the first report of behavioral and physiological differences between EPN species that conform to documented variation in habitat preference. Understanding the mechanisms by which EPNs adapt to a particular habitat could have practical applications by revealing how to screen existing populations, modify gene expression, and/or change habitat properties in ways that extend the geographic boundaries of otherwise promising native or introduced EPN species.

## Author contributions

FE and LD conceived and conducted all bioassays, NK perfomed proteomic analysis. All authors contributed to writing the manuscript.

## Funding

This research was supported by award number 525 from the Citrus Research and Development Foundation.

### Conflict of interest statement

The authors declare that the research was conducted in the absence of any commercial or financial relationships that could be construed as a potential conflict of interest.

## References

[B1] Abou-Setta M. M. Duncan L. W. (1998). Attraction of *Tylenchulus semipenetrans* and *Meloidogyne javanica* to salts *in vitro*. Nematropica 28, 49–59.

[B2] Beavers J. B. McCoy C. W. Kaplan D. T. (1983). Natural enemies of subterranean *Diaprepes-abbreviatus* (coleoptera, curculionidae) larvae in Florida. Environ. Entomol. 12, 840–843.

[B3] Bradford M. M. (1976). A rapid and sensitive method for the quantitation of microgram quantities of protein utilizing the principles of protein-dye binding. Anal. Biochem. 72, 248–252. 942051 10.1016/0003-2697(76)90527-3

[B4] Campos-Herrera R. Ali J. G. Díaz B. M. Duncan L. W. (2013b). Analyzing spatial patterns linked to the ecology of herbivores and their natural enemies in the soil. Front. Plant Sci. 4:378. 10.3389/fpls.2013.00378 24137165 PMC3786222

[B5] Campos-Herrera R. El-Borai F. E. Duncan L. W. (2016). Entomopathogenic nematode food web assemblages in Florida natural areas. Soil Biol. Biochem. 93, 105–114. 10.1016/j.soilbio.2015.10.022

[B6] Campos-Herrera R. El–Borai F. E. Ebert T. A. Schumann A. Duncan L. W. (2014). Management to control citrus greening alters the soil food web and severity of a pest–disease complex. Biol. Control 76, 41–51. 10.1016/j.biocontrol.2014.04.012

[B7] Campos-Herrera R. Gutiérrez C. (2009). Screening Spanish isolates of steinernematid nematodes for use as biological control agents through laboratory and greenhouse microcosm studies. J. Invertebr. Pathol. 100, 100–105. 10.1016/j.jip.2008.11.009 19073191

[B8] Campos-Herrera R. Johnson E. G. El–Borai F. E. Stuart R. J. Graham J. H. Duncan L. W. (2011). Long–term stability of entomopathogenic nematode spatial patterns measured by sentinel insects and real-time PCR assays. Ann. Appl. Biol. 158, 55–68. 10.1111/j.1744-7348.2010.00433.x

[B9] Campos-Herrera R. Pathak E. El-Borai F. E. Schumann A. Abd–Elgawad M. M. M. Duncan L. W. (2013c). New citriculture system suppresses native and augmented entomopathogenic nematodes. Biol. Control 66, 183–194. 10.1016/j.biocontrol.2013.05.009

[B10] Campos-Herrera R. Pathak E. El-Borai F. E. Stuart R. J. Gutiérrez C. Rodríguez-Martín J. A. (2013a). Geospatial patterns of soil properties and the biological control potential of entomopathogenic nematodes in Florida citrus groves. Soil Biol. Biochem. 66, 163–174. 10.1016/j.soilbio.2013.07.011

[B11] Choe K. P. (2013). Physiological and molecular mechanisms of salt and water homeostasis in the nematode *Caenorhabditis elegans*. Am. J. Physiol. Regul. Integr. Comp. Physiol. 305, 175–186. 10.1152/ajpregu.00109.2013 23739341

[B12] Duncan L. W. Dunn D. G. McCoy C. W. (1996). Spatial patterns of entomopathogenic nematodes in microcosms: implications for laboratory experiments. J. Nematol. 28, 252–258. 19277142 PMC2619690

[B13] Duncan L. W. Graham J. H. Dunn D. C. Zellers J. McCoy C. W. Nguyen K. (2003). Incidence of endemic entomopathogenic nematodes following application of *Steinernema riobrave* for control of *Diaprepes abbreviatus*. J. Nematol. 35, 178–186. 19265992 PMC2620623

[B14] Duncan L. W. Graham J. H. Zellers J. Bright D. Dunn D. C. El-Borai F. E.. (2007). Food web responses to augmenting the entomopathogenic nematodes in bare and animal manure–mulched soil. J. Nematol. 39, 176–189. 19259487 PMC2586487

[B15] Duncan L. W. McCoy C. W. (2001). Hydraulic lift increases herbivory by *Diaprepes ahhreviatus* larvae and persistence of *Steinernema riobrave* in dry soil. J. Nematol. 33, 142–146. 19266011 PMC2638139

[B16] Duncan L. W. Stuart R. J. El-Borai F. E. Campos-Herrera R. Pathak E. Graham J. H. (2013). Modifying orchard planting sites conserves entomopathogenic nematodes, reduces weevil herbivore and increases citrus tree growth, survival and fruit yield. Biol. Control 64, 26–36. 10.1016/j.biocontrol.2012.09.006

[B17] Dyballa N. Metzger S. (2009). Fast and sensitive colloidal coomassie G-250 staining for proteins in polyacrylamide gels. J. Vis. Exp. 893, 47–59. 10.3791/1431 PMC3149902 19684561

[B18] El-Borai F. E. Campos-Herrera R. Stuart R. J. Duncan L. W. (2011). Substrate modulation, group effects and the behavioral responses of entomopathogenic nematodes to nematophagous fungi. J. Invertebr. Pathol. 106, 347–356. 10.1016/j.jip.2010.12.001 21145324

[B19] El-Borai F. E. Duncan L. W. (2007). Suppression of *Diaprepes abbreviatus* in potted citrus by combinations of entomopathogenic nematodes with different lifespans. Nematropica 37, 33–41.

[B20] El-Borai F. E. Stuart R. J. Campos-Herrera R. Pathak E. Duncan L. W. (2012). Entomopathogenic nematodes, root weevil larvae, and dynamic interactions among soil texture, plant growth, herbivory, and predation. J. Invertebr. Pathol. 109, 134–142. 10.1016/j.jip.2011.10.012 22056274

[B21] Erkut C. Vasilj A. Boland S. Habermann B. Shevchenko A. Kurzchalia T. V. (2013). Molecular strategies of the *Caenorhabditis elegans* dauer larva to survive extreme desiccation. PLoS ONE 8:82473. 10.1371/journal.pone.0082473 24324795 PMC3853187

[B22] Evans A. A. F. Perry R. N. (2009). Survival mechanisms, in Root-knot Nematodes, eds Perry R. N. Moens M. Starr J. L. (Wallingford, CT: CABI), 201–219.

[B23] Foruney C. F. Brandl D. G. (1992). Control of humidity in small controlled-environment chambers using glycerol-water solution. Hort. Technol. 2, 52–54.

[B24] Gaugler R. (1999). Matching nematode and insect to achieve optimal field performance, in Optimal Use of Insecticidal Nematodes in Pest Management, ed Polavarapu S. (New Brundwick, NJ: Rutgers University), 9–14.

[B25] Hominick W. M. (2002). Biogeography in Entomopathhogenic Nematology, ed Gaugler R. (Wallinford; Oxon, UK: CABI), 115–143.

[B26] Hoy C. W. Grewal P. S. Lawrence J. L. (2008). Canonical correspondence analysis demonstrates unique soil conditions for entomopathogenic nematode species compared with other free-living nematode species. Biol. Control 46, 371–379. 10.1016/j.biocontrol.2008.06.001

[B27] Inada H. Ito H. Satterlee J. Sengupta P. Matsumoto K. Mori I. (2006). Identification of guanylyl cyclases that function in thermosensory neurons of *Caenorhabditis elegans*. Genetics 172, 2239–2252. 10.1534/genetics.105.050013 16415369 PMC1456394

[B28] Iwasa H. Maimaiti S. Kuroyanagi H. Kawano S. Inami K. Timalsina S.. (2013). Yes-associated protein homolog, YAP-1, is involved in the thermotolerance and aging in the nematode *Caenorhabditis elegans*. Exp. Cell Res. 319, 931–945. 10.1016/j.yexcr.2013.01.020 23396260

[B29] Jabbour R. Crowder D. W. Aultman E. A. Snyder W. E. (2011). Entomopathogen biodiversity increases host mortality. Biol. Control 59, 277–283. 10.1016/j.biocontrol.2011.07.016

[B30] Jenkins W. R. (1964). A rapid centrifugal-flotation technique for separating nematodes from soil. Plant Dis. Rept. 48, 492.

[B31] Kanga F. N. Waeyenberge L. Hauser S. Moens M. (2011). Distribution of entomopathogenic nematodes in Southern Cameroon. Invertebr. Pathol. 109, 41–51. 10.1016/j.jip.2011.09.008 21983478

[B32] Kaplan D. T. Davis E. L. (1990). Improved nematode extraction from carrot disk culture. J. Nematol. 22, 399–406. 19287736 PMC2619058

[B33] Kaspi R. Ross A. Hodson A. Stevens G. N. Kaya H. K. Lewis E. E. (2010). Foraging efficacy of the entomopathogenic nematode *Steinernema riobrave* in different soil types from California citrus groves. Appl. Soil Ecol. 45, 243–253. 10.1016/j.apsoil.2010.04.012

[B34] Keller A. Nesvizhskii A. L. Kolker E. Aebersold R. (2002). Empirical statistical model to estimate the accuracy of peptide identifications made by MS/MS and database search. J. Anal. Chem. 74, 5383–5392. 10.1021/ac025747h 12403597

[B35] Kung S. P. Gaugler R. Kaya H. K. (1990). Influnce of soil pH and oxygen on persistence of *Steinernema* spp. J. Nematol. 22, 440–445. 19287743 PMC2619085

[B36] Lu Z. Qin A. Qian K. Chen X. Jin W. Zhu Y.. (2010). Proteomic analysis of the host response in the bursa of Fabricius of chickens infected with Marek's disease virus. Virus Res. 153, 250–257. 10.1016/j.virusres.2010.08.010 20723570

[B37] Mackenzie J. M. Jr. Epstein H. F. (1980). Paramyosin is necessary for determination of nematode thick filament length *in vivo*. Cell 22, 747–755. 10.1016/0092-8674(80)90551-6 7193096

[B38] McCoy C. W. (1999). Arthropod pests of citrus roots, in Citrus Health Management, eds Timmer L. W. Duncan L. W. (St. Paul, MN: American Phytopathological Society), 149–156.

[B39] McCoy C. W. Shapiro D. I. Duncan L. W. Nguyen K. (2000). Entomopathogenic nematodes and other natural enemies as mortality factors for larvae of *Diaprepes abbreviatus* (Coleoptera: Curculionidae). Biol. Control 19, 182–190. 10.1006/bcon.2000.0852

[B40] Mekete I. Gaugler R. Nguyen K. B. Mandefro W. Tessera M. (2005). Biogeography of entomopathogenic nematodes in Ethiopia. Nematropica 35, 31–36.

[B41] Mwaitulo S. Haukeland S. Sæthre M.-G. Laudisoit A. Maerere A. P. (2011). First report of entomopathogenic nematodes from Tanzania and their virulence against larvae and adults of the banana weevil *Cosmopolites sordidus* (Coleoptera: Curculionidae). Int. J. Trop. Insect Sci. 31, 154–161. 10.1017/S1742758411000294

[B42] Nesvizhskii A. Keller A. Kolker E. Aebersold R. (2003). A statistical model for identifying proteins by tandem mass spectrometry. J. Anal. Chem. 75, 4646–4658. 10.1021/ac0341261 14632076

[B43] Nguyen K. B. (2007). Methodology, morphology and identification, in Entomopathogenic Nematodes: Systematics, Phylogeny and Bacterial Symbionts, Vol. 5, Nematology Monographs and Perspectives, eds Nguyen K. B. Hunt D. J. (Leiden; Boston, MA: Brill), 59–119.

[B44] Russell J. Vidal-Gadea A. G. Makay A. Lanam C. Pierce-Shimomura J. T. (2014). Humidity sensation requires both mechanosensory and thermosensory pathways in *Caenorhabditis elegans*. Proc. Natl. Acad. Sci. U.S.A. 111, 8269–8274. 10.1073/pnas.1322512111 24843133 PMC4050571

[B45] Schumann A. W. Syvertsen J. P. Morgan K. T. (2012). Implementing advanced citrus production systems in Florida – early results. Proc. Fla. State Hort. Soc. 122, 108–113.

[B46] Spiridonov S. E. Reid A. P. Podrunka K. Subbotin S. A. Moens M. (2004). Phylogenetic relationships within the genus *Steinernema* (Nematoda: Rhabditida) as inferred from analyses of sequences of the ITSI-5.8S-ITS2 región of rDNA and morphological features. Nematology 6, 547–566. 10.1163/1568541042665304

[B47] Tsou M. F. Hayashi A. DeBella L. R. McGrath G. Rose L. S. (2002). LET-99 determines spindle position and is asymmetrically enriched in response to PAR polarity cues in *C. elegans* embryos. Development 129, 4469–4481. 10.3410/f.1004455.148616 12223405

[B48] Valadas V. Laranjo M. Mota M. Oliveira S. (2014). A survey of entomopathogenic nematode species in continental Portugal. J. Helminth. 88, 327–341. 10.1017/S0022149X13000217 23590880

[B49] Wang H. Luan J. Dong H. Qian H. Cong B. (2014). Natural occurrence of entomopathogenic nematodes in Liaoning (Northeast China) J. Asia Pacif. Entomol. 17, 399–406. 10.1016/j.aspen.2014.03.005

[B50] Woodring J. L. Kaya H. K. (1988). Steinernematid and Heterorhabditid Nematodes: A Handbook of Techniques. Southern Cooperative Series Bulletin. Fayetteville: Arkansas Agricultural Experiment Station, 30.

[B51] Zadji L. Baimey H. Afouda L. Houssou F. G. Waeyenberge L. de Sutter N. (2013). First record on the distribution of entomopathogenic nematodes in southern Benin. Russ. J. Nematol. 21, 117–130.

